# Dietary Histidine Supplementation Maintained Amino Acid Homeostasis and Reduced Hepatic Lipid Accumulation of Juvenile Largemouth Bass, *Micropterus Salmoides*

**DOI:** 10.1155/2022/4034922

**Published:** 2022-10-25

**Authors:** Hualiang Liang, Gangchun Xu, Pao Xu, Jian Zhu, Songlin Li, Mingchun Ren

**Affiliations:** ^1^Key Laboratory of Integrated Rice-Fish Farming Ecology, Ministry of Agriculture and Rural Affairs, Freshwater Fisheries Research Center, Chinese Academy of Fishery Sciences, Wuxi 214081, China; ^2^Research Centre of the Ministry of Agriculture and Rural Affairs on Environmental Ecology and Fish Nutrition, Shanghai Ocean University, Shanghai 201306, China

## Abstract

This 56-day research aimed to evaluate the recommended histidine requirement and the influence of dietary histidine levels on the protein and lipid metabolism of juvenile largemouth bass (*Mieropterus salmoides*). The initial weight of the largemouth bass was 12.33 ± 0.01 g, which was fed with six graded levels of histidine. The results showed that appropriate dietary histidine had a positive effect on growth, with a higher specific growth rate, final weight, weight gain rate, protein efficiency rate, and a lower feed conversion rate and feed intake rate being observed in 1.08-1.48% dietary histidine groups. Furthermore, the mRNA levels of GH, IGF-1, TOR, and S6 showed an increasing trend first and then declined, similar to the trend of the growth and protein content of the whole body composition. Meanwhile, dietary histidine levels could be sensed by the AAR signaling pathway, representing as downregulation of core genes of AAR signaling pathway with the increased dietary histidine levels, including GCN2, eIF2*α*, CHOP, ATF4, and REDD1. In addition, increased dietary histidine levels decreased the lipid content of the whole body and the liver by upregulating the mRNA levels of core genes of the PPAR*α* signaling pathways, including PPAR*α*, CPT1, L-FABP, and PGC1*α*. However, increased dietary histidine levels downregulated the mRNA levels of core genes of the PPAR*γ* signaling pathways such as PPAR*γ*, FAS, ACC, SREBP1, and ELOVL2. These findings were also supported by the positive area ratio of hepatic oil red O staining and the TC content of plasma. According to the specific growth rate and feed conversion rate, the recommended histidine requirement of juvenile largemouth bass was 1.26% of the diet (2.68% of dietary protein) by regression lines calculated using a quadratic model. In general, histidine supplementation promoted protein synthesis and lipid decomposition and reduced lipid synthesis by activating the TOR, AAR, PPAR*α*, and PPAR*γ* signaling pathways, which provided a new perspective to solve the fatty liver problem of largemouth bass by nutritional means.

## 1. Introduction

There are many factors affecting the growth of farmed fish, and the food availability is considered the most important one [1]. Feed cost is usually the highest recurring cost in the total cost of fish culture. Hence, controlling the balance of feed nutrition while reducing feed cost is the key to commercial success of aquaculture [2]. The protein level of a fish is very important to its overall nutrition. The importance of nutritional protein has been established by a gradient culture experiment of dietary essential amino acids that observed their effects on growth performance [3]. Amino acids are substrates for protein synthesis and key regulators of many important metabolic pathways. Thus, they are essential nutrients for fish [4], and deficiency of essential amino acids could result in the inadequate utilization of dietary protein, which leads to poor weight gain, feed efficiency and disease resistance [5].

Since aquatic animals have poor capacities for the synthesis of endogenous histidine, it is considered one of the essential amino acids of fish [6], playing an indispensable role in regulating growth, protein synthesis, hemoglobin synthesis, tissue formation, immune response, etc. [5, 7, 8]. In previous studies, histidine requirements of numerous fishes have been identified, such as Indian catfish (*Heteropneustes fossilis* (Bloch)) (1.35% of dietary protein) [2], grass carp (*Ctenopharyngodon idella*) (3.2% of dietary protein) [6], Nile tilapia (*Oreochromis niloticus*) (3.1% of dietary protein) [8], red drum (*Sciaenops ocellatus*) (1.6% of dietary protein) [9], Jian carp (*Cyprinus carpio* var. Jian) (2.38% of dietary protein) [10], and large yellow croaker (*Pseudosciaena crocea* R) (1.88-2.08% of dietary protein) [11]. Indeed, when histidine is deficient, the growth performance of many fish species would be inhibited. Therefore, a concerted effort is needed to determine the optimal dietary histidine requirements apart from the true protein requirements of farmed fish.

There are two known signaling pathways that can sense amino acid content, one is target of rapamycin (TOR) signaling system that senses amino acid abundance, and the other is amino acid response (AAR) signaling pathway that senses amino acid balance [12]. Some studies have shown that when amino acids are abundant, TOR senses amino acid levels and is activated to enhance protein synthesis by affecting the downstream core genes AKT, S6, and 4EBBP1, however, when amino acid is deficient, TOR signaling pathway is inhibited, and AAR signaling is activated and regulating the expression of core genes GCN2, eIF2, CHOP, ATF4, and REDD1, which are involved in the synthesis and breakdown of proteins at the systemic level and maintaining the amino acid homeostasis [13–18]. Previous studies have confirmed that grass carp and blunt snout bream could sense dietary histidine levels by TOR signialing pathway [13, 19, 20]. However, researches on AAR signaling pathway sensing dietary histidine levels were insufficient. Nevertheless, in other studies of amino acids, largemouth bass (*Micropterus salmoides*) was found to be able to sense the levels of dietary methionine and lysine to regulate protein synthesis and maintain the balance of amino acids through TOR and AAR signaling pathways [17, 18]. Similar results have also been reported in Indian major carp [2], fingerling Indian catfish [7], juvenile blunt snout bream [13], and Catla (*Catla catla*) (Hamilton) [21]. Furthermore, many studies have found that amino acid/protein is also involved in regulating lipid metabolism in fish. For example, arginine, leucine, and tryptophan were found to regulate lipid metabolism in blunt snout bream [22–24]. Protein and lysine were also found to regulate lipid metabolism in grass carp [25, 26], and protein and methionine were found to regulate lipid metabolism in juvenile genetically-improved farmed tilapia (GIFT) (*Oreochromis niloticus*) [27, 28]. These processes might be related with the peroxisome proliferator-activated receptor *α* (PPAR*α*) and peroxisome proliferator-activated receptor *γ* (PPAR*γ*) signaling pathways [29, 30].

Largemouth bass is native to freshwater rivers and large lakes in America, especially the Great Lakes in the United States. It has been widely raised around the world because of its tender meat and delicious taste, and loved by consumers and sold well in the international market, known as “Freshwater grouper”. As far as China is concerned, production of largemouth bass has reached 619, 000 tons in 2020 [31]. Hence, researchers are paying more and more attention to the nutritional requirements of largemouth bass, in an effort to provide theoretical basis for the development of its compound feed. Some amino acid requirements for largemouth bass have been identified already such as lysine [32, 33] and threonine [34]. However, research on histidine requirement is still blank. Meanwhile, carnivorous fish are more sensitive to dietary carbohydrate levels, which can easily cause the disorder of carbohydrate and lipid metabolism [35]. Largemouth bass, as a typical carnivorous fish, often encounters metabolic disorders, resulting in hepatic lipid accumulation and even liver function lesions, thus inhibiting growth and leading to death [36]. In particular, the fatty liver problems of largemouth bass have been troubling the growth and nutrition metabolism of the fish. In recent years, amino acids' important role in regulating lipid metabolism has been largely studied [26, 28, 32], however, the regulation of histidine on lipid metabolism of largemouth bass has not been studied. Therefore, this 56-day research was carried out to evaluate the recommended histidine requirement and the regulation of dietary histidine levels on the metabolism of protein and lipid of juvenile largemouth bass.

## 2. Experimental Procedure

### 2.1. Ethical Statement

The experimental protocol followed that of the Institutional Animal Care and Ethics Committee of Nanjing Agricultural University, Nanjing, China. [Permit number: SYXK (Su) 2011-0036] and the Standardization Administration of China protocols and guidelines (GB/T 35892-2018).

### 2.2. Diets

Six different histidine levels were designed according to isonitrogenous and isoenergetic principles. These levels were 0.71%, 0.89%, 1.08%, 1.26%, 1.48%, and 1.67%, respectively. Fish meal, rapeseed meal, and soybean meal provided main protein of the feed, and fish oil provided the main lipid. The amino acid mixes were calculated and added to the feed based on dietary protein content (Tables 1 and 2). All ingredients were crushed and passed through an 80 *μ*m mesh sieve, well mixed according to the experimental formula, then made into 1 mm sized pellet feed using a SJPS56x2 bulking machine (Jiangsu Muyang Holdings Co., LTD). The wet feed was then dried at 45°C and stored in a refrigerator at -20°C.

### 2.3. Breeding

All experimental fish were temporarily kept in pond cages for 15 days to acclimate to the environment. Then, juvenile largemouth bass (12.33 ± 0.01 g) with a healthy physique, sufficient vitality, and similar specifications were randomly divided and stocked into 18 experimental cages, with 20 fish in each cage. All the fishes were fed two times daily (7:00 and 17:00) based on apparent satiety criteria throughout the breeding experiment stage. During this experiment, aquaculture water parameters were controlled within the following range: 28 ± 2°C (water temperature), 7-7.5 (pH), > 6.0 mg/L (dissolved oxygen), and < 0.1 mg/L (total ammonia nitrogen).

### 2.4. Samples

After the breeding experiment, the fishes were all starved for 24 h, and then each cage of fish was bulk-weighed and counted. Meanwhile, blood was drawn from the tail vein and centrifuged at 3,000 rpm (10 min, 4°C) to plasma. Then, the fishes were dissected to obtain liver samples; some of which were stored in sample sacks for the content analysis of liver; some were stored in cryopreservation tubes for gene expression analysis, and some were fixed in 4% paraformaldehyde for oil red O staining analysis.

### 2.5. Experimental Analysis

#### 2.5.1. Proximate Analysis and Biochemical Analysis

The proximate analysis of the raw materials, diets, and whole-body composition and the lipid content of liver was conducted using the established methods of AOAC [37]. The principal detection equipment and methods are summarized in Table 3. The amino acid composition of ingredients and feed was evaluated in accordance with previous studies [38]. Furthermore, the total triglyceride (TG) and total cholesterol (TC) levels of plasma were also analyzed by a biochemical analyzer (Mindray Medical International Ltd., China) (Table 3).

#### 2.5.2. Oil Red O Staining

The fresh liver samples were fixed in 4% paraformaldehyde for no less than 48 hours. Then, the tissue was dehydrated and embedded with optimal cutting temperature compound. This was then sliced at a thickness of 8-10 *μ*m through a leica RM 2135 slicing machine (Leica Company, Wetzlar, Germany). The tissue sections were then stained with oil red O as follows: (a) drying tissue sections at room temperature for 10 minutes; (b) incubating tissue sections at 75% alcohol for 10 seconds and oil red O working fluid for 10-15 minutes (away from light); (c) differentiation using 75% alcohol for 2 seconds; (d) washing with water for 1 minute and redying with hematoxylin; (e) washing with water and an ammonia solution until the section was blue; (f) draining the water and sealing the tissue sections with glycerin and gelatin.

#### 2.5.3. Gene Expression Analysis

The related genes mRNA levels of protein synthesis, amino acid response, and lipid metabolism were determined by qRT-PCR analysis, which was referred to our previous study [39]. First, RNA extraction was operated by the TRIzol method (Vazyme Biotech Co., Ltd, China). Second, the thermo Scientific NanoDrop-2000 Spectrophotometers (Thermofisher Scientific, USA) was used for testing the quantity and quality of RNA. Finally, qRT-PCR was analyzed using a CFX96 Touch Real-Time PCR Detection System (Bio-Rad, USA). The program was reverse transcription for 5 minutes at 42°C, predegeneration for 10 seconds at 95°C, 40 cycles of denaturation for 5 seconds at 95°C, and 34 seconds at 60°C (annealing temperature). The melting curve was analyzed by elevating the temperature from 65°C to 95°C while monitoring fluorescence. The primers for the analysis of mRNA levels were shown in Table 4. The Glyceraldehyde-3-phosphate dehydrogenase (GAPDH) was selected as internal reference gene, and the levels of mRNA were calculated from the standard curve, normalized against GAPDH and quantified using a relative standard curve method [40].

### 2.6. Statistical Analysis

The area of oil red O staining in the tissue section was analyzed by Image-Pro 6.0 software, which calculated the positive area, tissue area, and the proportion of the positive area. Furthermore, SPSS 16.0 software was also used for analyzing data by one-way analysis of variance (ANOVA) and Tukey's multiple comparisons test. All the analyzed data were presented as the means with S.E.M. *P* < 0.05, which was considered to be a significant difference.

After comparing the estimation coefficient (R^2^) among the linear regression model (SGR, R^2^ = 0.151; FCR, R^2^ = 0.135), logarithmic regression model (SGR, R^2^ = 0.277; FCR, R^2^ = 0.208), quadratic linear regression model (SGR, R^2^ = 0.717; FCR, R^2^ = 0.721), and broken regression model (SGR, R^2^ = 0.552; FCR, R^2^ = 0.551), the quadratic regression model was selected to determine the optimum dietary histidine requirement of largemouth bass.

## 3. Results

### 3.1. Growth Performance

**Figure 1:**
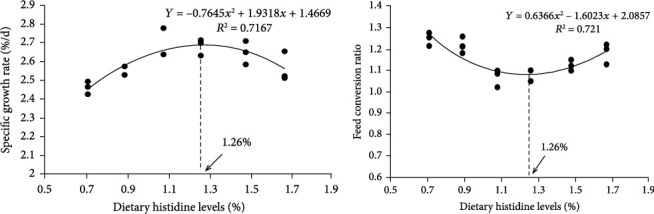
Quadratic regression analysis of specific growth rate (SGR, %/day) and feed conversion rate (FCR) against graded different levels of dietary histidine.

Appropriate histidine levels had a positive effect on growth. Histidine adding could significantly improve the final weight, specific growth rate, weight gain rate, and protein efficiency rate as well as significantly lower the feed conversion rate and feed intake rate at the 1.08-1.48% dietary histidine levels (*P* < 0.05). However, there were no significant differences found in survival rate, condition factor, viscerosomatic index, and hepatosomatic index (*P* > 0.05) (Table 5). Therefore, based on the specific growth rate and feed conversion rate, the dietary histidine requirement of juvenile largemouth bass was 1.26% of diet (2.68% of dietary protein) as determined (Figure 1).

### 3.2. Whole-Body Composition and Serum Biochemical Indices

**Figure 2:**
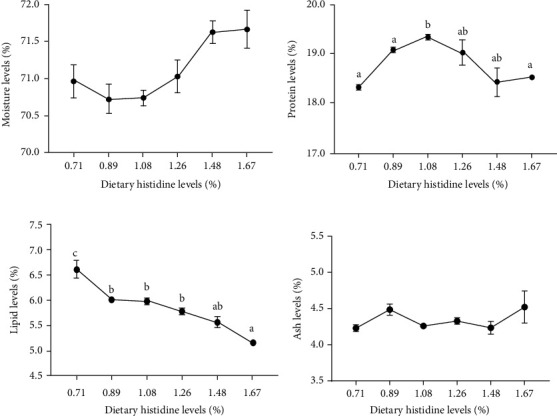
The whole body composition of juvenile largemouth bass fed with different levels of dietary histidine. Data are expressed as means with S.E.M.; value with different superscripts are significantly different (*P* < 0.05).

**Figure 3:**
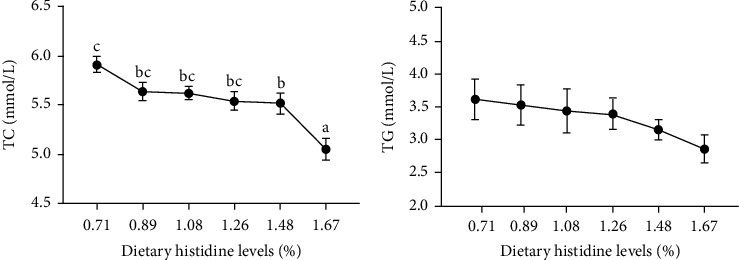
Serum biochemical indices of juvenile largemouth bass fed with different levels of dietary histidine. Data are expressed as means with S.E.M.; value with different superscripts are significantly different (*P* < 0.05).

The fish fed with the 1.08% dietary histidine showed highest protein content (*P* < 0.05) (Figure 2(b)). Meanwhile, the lipid levels showed a continuing trend of decline with the increase of histidine supplementation (*P* < 0.05) (Figure 2(c)). And the plasma TC levels also showed a trend of decline with the increase of histidine supplementation (*P* < 0.05) (Figure 3(a)). However, there were no significant differences found in the ash and moisture levels of whole-body composition and plasma TG (*P* > 0.05) (Figures 2(a) and 2(d) and Figure 3(b)).

### 3.3. TOR and GH-IGF-1 Signaling Pathway

**Figure 4:**
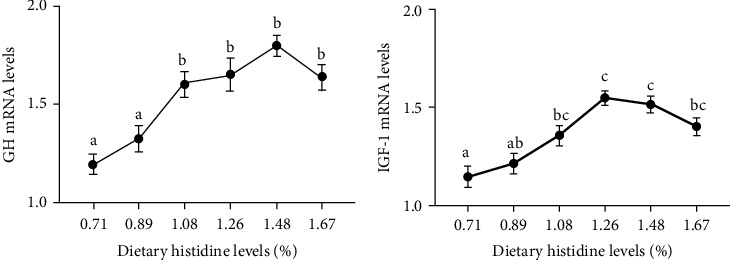
The relative expressions of GH-IGF-1 axis in liver of juvenile largemouth bass fed with different levels of dietary histidine. Data are expressed as means with S.E.M.; value with different superscripts are significantly different (*P* < 0.05).

**Figure 5:**
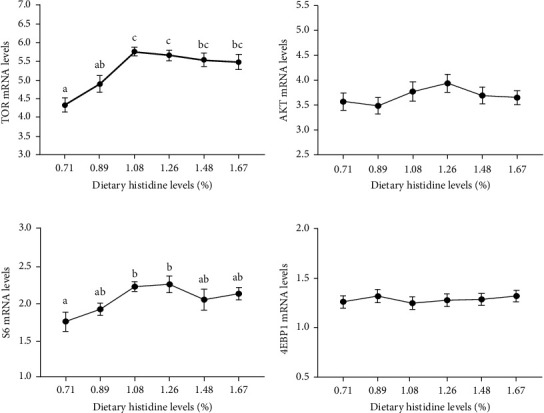
The relative expressions of TOR signaling pathway in liver of juvenile largemouth bass fed with different levels of dietary histidine. Data are expressed as means with S.E.M.; value with different superscripts are significantly different (*P* < 0.05).

The 1.08-1.67% dietary histidine levels significantly improved the mRNA levels of growth hormone (GH), insulin-like growth factor-1 (IGF-1), and TOR (*P* < 0.05) (Figures 4(a) and 4(b) and Figure 5(a)). Furthermore, the 1.08 and 1.26% dietary histidine levels significantly increased the ribosomal protein S6 (S6) mRNA levels (*P* < 0.05) (Figure 5(c)). However, no significant effects on the mRNA levels of pukaryotic initiation factor 4E binding protein 1 (4EBP1) and protein kinase B (AKT) were found in juvenile largemouth bass fed with different dietary histidine levels (*P* > 0.05) (Figures 5(b) and 5(d)).

### 3.4. AAR Signaling Pathway

**Figure 6:**
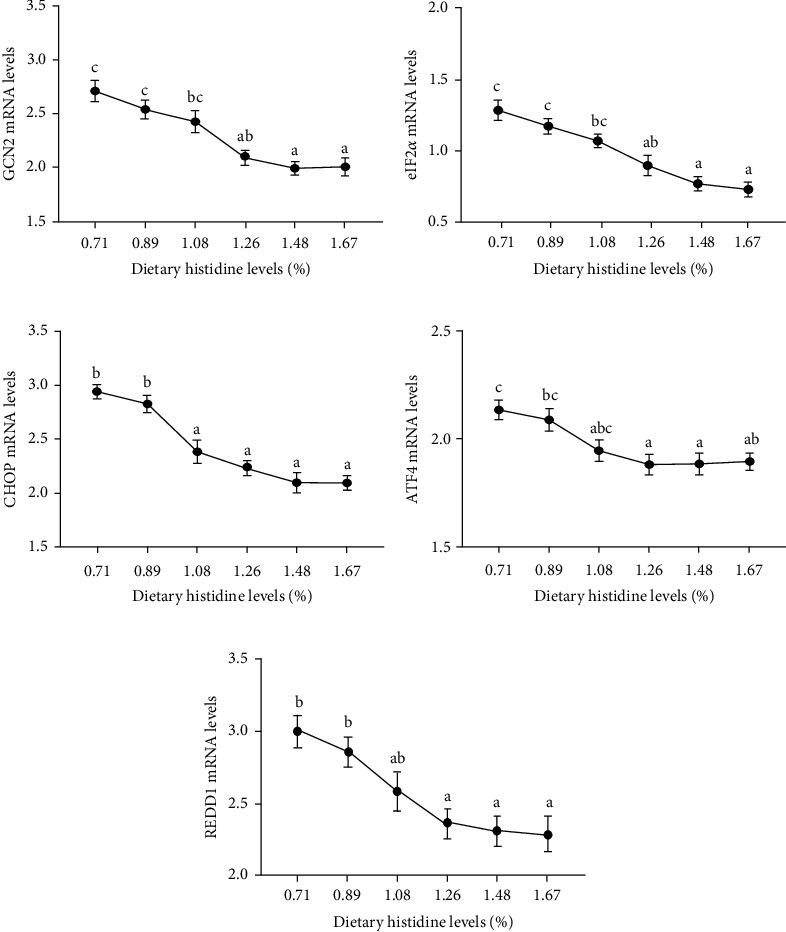
The relative expressions of AAR signaling pathway in liver of juvenile largemouth bass fed with different levels of dietary histidine. Data are expressed as means with S.E.M.; value with different superscripts are significantly different (*P* < 0.05).

The mRNA levels of general control nonderepressible 2 kinase (GCN2) had a continuing trend of decline with the increase of dietary histidine levels (*P* < 0.05) (Figure 6(a)). Furthermore, the mRNA levels of downstream genes of GCN2 signaling pathway, including eukaryotic initiation factor 2*α* (eIF2*α*), C/EBP-homologous protein (CHOP), transcription factor 4 (ATF4), and DNA damage responses 1 (REDD1), were also decreased with the increase of dietary histidine levels (*P* < 0.05) (Figures 6(b–e)).

### 3.5. PPAR*α* and PPAR*γ* Signaling Pathways

**Figure 7:**
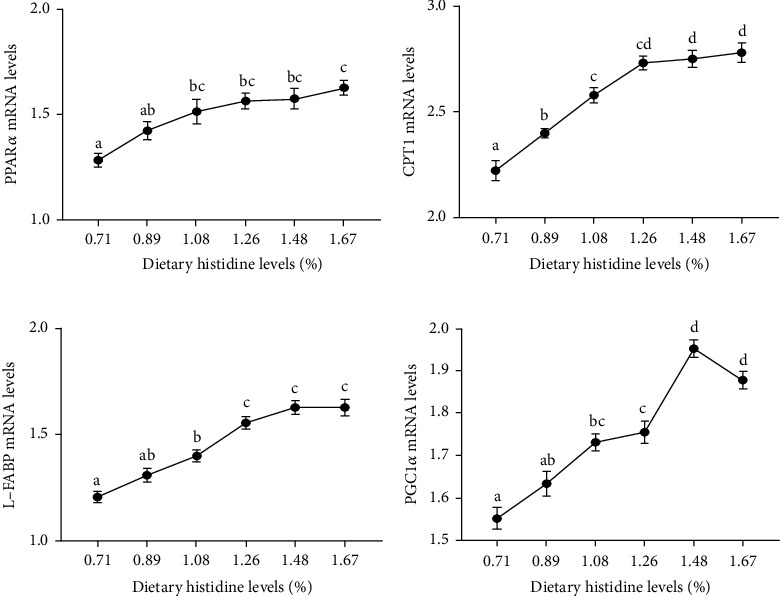
The relative expressions of PPAR*α* signaling pathway in liver of juvenile largemouth bass fed with different levels of dietary histidine. Data are expressed as means with S.E.M.; value with different superscripts are significantly different (*P* < 0.05).

**Figure 8:**
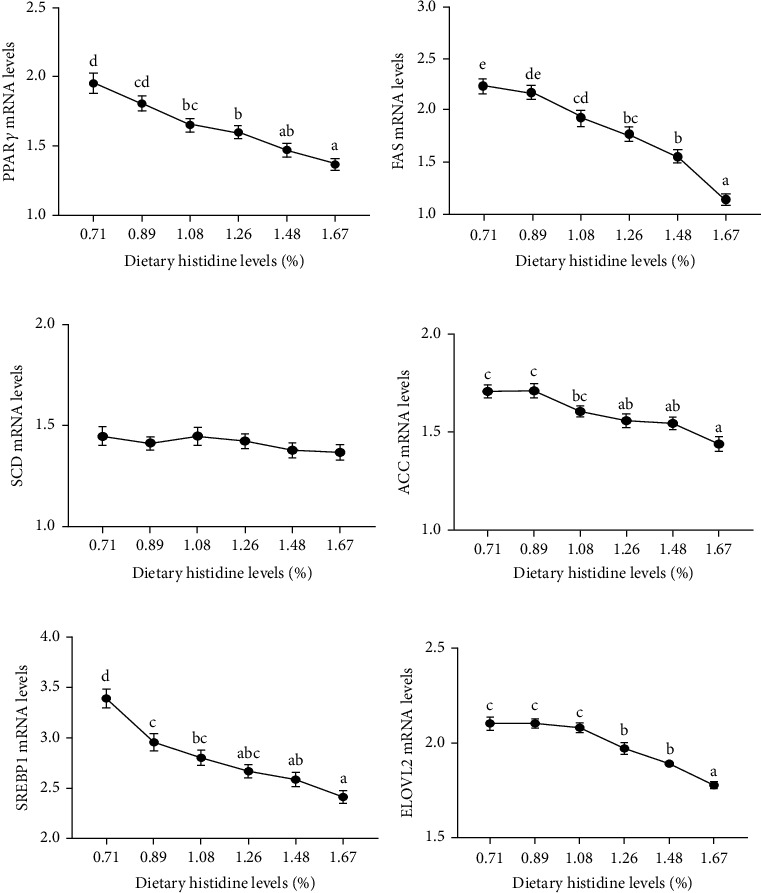
The relative expressions of PPAR*γ* signaling pathway in liver of juvenile largemouth bass fed with different levels of dietary histidine. Data are expressed as means with S.E.M.; value with different superscripts are significantly different (*P* < 0.05).

The mRNA levels of PPAR*α* were increased with the increasing dietary histidine levels (*P* < 0.05) (Figure 7(a)). Furthermore, the mRNA levels of downstream genes of PPAR*α* signaling pathway, including carnitine palmitoyl transferase-1 (CPT1), Liver fatty acid binding protein (L-FABP), and peroxisome proliferator activator receptor *γ* coactivator-1*α* (PGC1*α*), were also increased with the increasing dietary histidine levels (*P* < 0.05) (Figures 7(b–d)). Moreover, the mRNA levels of PPAR*γ* had an opposite trend with the mRNA levels of PPAR*α* (*P* < 0.05) (Figure 8(a)). Furthermore, the mRNA levels of the downstream genes of the PPAR*γ* signaling pathway, including fatty acid synthase (FAS), Acetyl-CoA carboxylase (ACC), sterol-regulatory element binding protein-1 (SREBP1), and elongation of very long-chain fatty acids 2 (ELOVL2), were significantly decreased in the fish fed different dietary histidine levels (*P* < 0.05) (Figures 8(b) and 8(d–f)). However, different dietary histidine levels showed no significant effect on the mRNA levels of stearoyl-CoA desaturase (SCD) (*P* > 0.05) (Figure 8(c)).

### 3.6. The Hepatic Oil Red O Stain and Lipid Content

**Figure 9:**
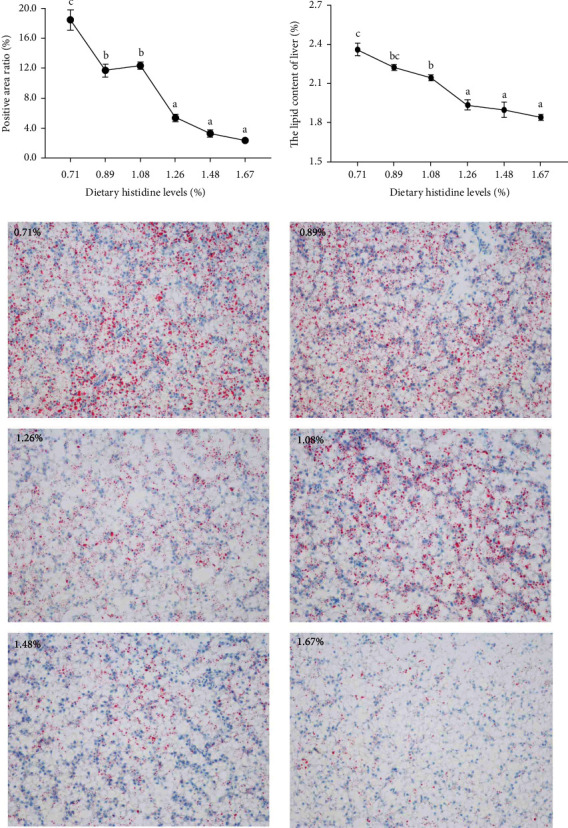
The hepatic oil red O staining and lipid content of juvenile largemouth bass fed with different levels of dietary histidine. Data are expressed as means with S.E.M.; value with different superscripts are significantly different (*P* < 0.05).

The positive area ratio of hepatic oil red O staining showed a significant lower trend with the increase of dietary histidine levels (*P* < 0.05). Furthermore, the lipid content of liver also showed a similar trend with positive area ratio of oil red stain (*P* < 0.05) (Figure 9).

## 4. Discussion

In this study, dietary histidine deficiency inhibited the growth of largemouth bass, which showed a decrease observed in the final weight, weight gain rate, specific growth rate, protein efficiency rate, and an increase observed in the feed conversion rate and feed intake rate. The results were similar to the reports in many kinds of fish such as grass carp [6], Nile tilapia [8], red drum [9], Jian carp [10], large yellow croaker [11], blunt snout bream [13], etc. In the lowest level of dietary histidine (0.71%), no overt deficiency signs, other than reduced growth, were observed in this study, which was similar to the fingerling Indian catfish [7]. However, cataract symptoms were observed in fish fed the histidine-deficient diet in red drum [9], and there was also a close relationship between dietary histidine level and the incidence of cataracts in Atlantic salmon (*Salmo salar* L.) [41]. It is reported that N-acetylhistidine could be synthesized in the lens and was formed by the acetylation of histidine, which can happens rapidly in the lens and relieve cataracts [42]. Hence, histidine might play an important role in preventing cataract in aquatic animals. Different from this study, this could be due to the lower histidine levels in the experiment of juvenile red drum [9]. Based on the feed conversion rate and specific growth rate, the appropriate dietary histidine level was 1.26% of diet (2.68% of dietary protein) by quadratic regression analysis, which was similar to the requirements reported in grass carp (3.2% of dietary protein) [6], Nile tilapia (3.1% of dietary protein) [8], Jian carp (2.38% of dietary protein) [10], and stinging catfish (2.48% of dietary protein) [43]. However, it was higher than the reports in Indian catfish (1.35% of dietary protein) [7] and red drum (1.6% of dietary protein) [9]. These differences might be due to the differences of fish size, ingredients types, protein sources, feed palatability, fish species, and environmental conditions. Furthermore, the GH-IGF-1 axis had an improtant role in regulating somatic growth [44]. In this study, the mRNA levels of GH and IGF-1 were significantly upregulated by 1.08%-1.67% dietary histidine levels, which partly explained the reason that histidine supplementation could improve the growth performance of largemouth bass.

There are two known signaling pathways that can sense amino acid content, TOR signaling system, which senses amino acid abundance, and AAR signaling pathway, which senses amino acid balance [12]. In this study, appropriate histidine levels significantly increased the TOR and S6 gene expression and inhibited the expression of 4EBP1 gene. A similar trend was observed for growth performance and the protein content of the whole body. Previous studies have confirmed that appropriate histidine levels could activate TOR signaling pathway in grass carp and blunt snout bream, which affected the downstream gene expression to regulate protein synthesis [13, 19, 20]. Furthermore, our previous studies showed that adequate AA supplementation for grass carp and blunt snout bream could significantly activated the TOR signaling pathway and improve growth performance, protein synthesis and protein deposition [26, 38]. The AAR signaling pathway, another nutrient-sensing signaling pathway, is responsible for sensing amino acid deficiency and thus maintaining homeostasis [12, 16–18]. Furthermore, the AAR signaling pathway was activated by upregulating the expression genes of the AAR signaling pathway including GCN2, eIF2*α*, ATF4, CHOP, and REDD1. The expression of these genes tended to a decrease tend with increased dietary histidine levels, which was the opposite trend with the mRNA levels of TOR and S6. The present study showed that fish also have a dynamic regulatory mechanism of nutrition; AA deficiencies may activate the AAR signaling pathways to elevate uncharged tRNAs levels and further binds to GCN2, which depressed whole amino acid translation and synthesis under nutrient stress [16–18]. However, adequate AA levels could facilitate growth and protein synthesis by the TOR signaling pathway [23, 45]. All of these results show that the two signaling pathways respond together to maintain the body in a state of optimal growth during nutritional status changes [17, 18]. These results are strongly supported by extensive research literature on the TOR/AAR-sensing pathways in rainbow trout (*Oncorhynchus mykiss*) [46], turbot (*Scophthalmus maximus* L.), [47] and largemouth bass [17, 18]. Hence, dietary histidine levels can be sensed by TOR and AAR signaling pathways by regulating the key genes expressions of TOR, S6, GCN2, eIF2*α*, ATF4, CHOP, and REDD1, which systematically regulate amino acid homeostasis in the fish.

Recently, amino acids have been widely identified as signaling molecules that regulate lipid metabolism in aquatic animals [22, 23]. PPAR*α*, as one of the PPARs subtypes, is responsible for regulating *β* oxidation and promoting lipid metabolism [30]. In this study, the mRNA levels of PPAR*α* were increased with the increase of dietary histidine levels, which further downregulated the downstream target genes, including CPT1 and L-FABP. In Nile tilapia, fenofibrate activated PPAR*α* to show a hypolipidemic effect [48]. Furthermore, 200 mg/kg gemfibrozil (an agonist of PPAR*α*) activated PPAR*α* to promote lipid catabolism by improving the tolerance of Nile tilapia to a high-sugar diet and alleviating the negative effects caused by a high-sugar diet [49]. However, the deletion of PPAR*α* in mice downregulated the mRNA levels of lipid catabolism-related genes, and metabolism was blocked in mice fed high-lipid diet. This resulted in a marked increase in hepatic lipid deposition and even led to the eventual occurrence of obesity [50]. All of these results demonstrated that dietary histidine supplementation reduced lipid deposition by activating the PPAR*α* signaling pathway. PGC-1*α*, as a PPAR*γ* coactivator, is to regulate lipid metabolism [51]. In this study, the mRNA levels of PGC1*α* were upregulated by dietary histidine levels. It has been reported that PPAR*α* relies on PGC1*α* to effectively exert its lipid-lowering effect effectively [51]. Furthermore, the mRNA levels of PGC1*α* were also upregulated when PPAR*α* was activated, which confirmed that PGC1*α* is one of the key coactivators in the regulation of fatty acid catabolism by PPAR*α* in the cardiac metabolic stress response [52]. These results indicated that PGC1*α* might coordinate with PPAR*α* to induce the gene expression of fatty acid catabolism-related enzymes to regulate lipid metabolism. Furthermore, PPAR*γ* is another important regulator of lipid metabolism [29]. In this study, the PPAR*γ* mRNA levels were decreased with increasing dietary histidine levels. This further downregulated its downstream genes, including FAS, ACC, and ELOVL2. These results showed that dietary histidine deficiency might increase lipid synthesis by upregulation of the PPAR*γ*, FAS, ACC, and ELOVL2, and dietary histidine supplementation could inhibit lipid synthesis in a dose-dependent manner. The antiobesity effects of dietary-supplemented amino acids have been reported [53]. However, different from our study, the lipid content of whole-body composition was increased with the supplementary histidine levels in fingerling Indian catfish [7]. This difference might be due to the low level of histidine compared with our study. Moreover, it has been reported that SREBP1, as a transcription factor of fatty acid synthesis, can interact with PPAR*γ* to promote lipid synthesis [54]. In this study, the expression of SREBP1 showed a similar trend, indicating that SREBP1 might perform a function in activating PPAR*γ* to regulate lipid synthesis. The main mechanism of SREBP1 acts to control the production of endogenous ligand(s) of PPAR*γ* and coordinate these lipid-forming factors [55]. Generally speaking, all of the results described were also supported by liver lipid content, serum triglyceride levels, and the positive area of oil red O staining. Similar to our study, dietary lysine supplementation reduced lipid deposits in grass carp [26]. However, the higher lipid content was improved when amino acid supplementation was excessive or appropriate such as lysine in arginine in blunt snout bream [22] and freshwater catfish (*Mystus nemurus*) [56], methionine in rainbow trout [46]. Different dietary amino acid supply conditions might also have completely opposite consequences in different species [26]. The mechanism of this difference is still unclear and further investigation is needed.

## 5. Conclusion

**Figure 10:**
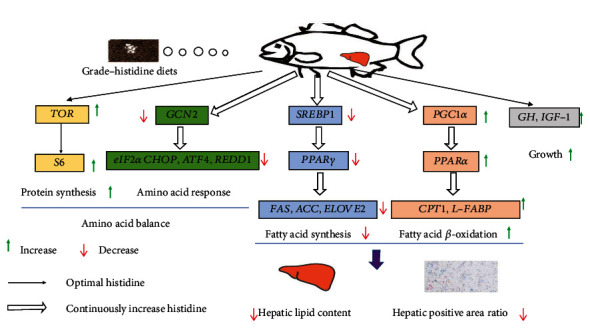
Scheme summarizing the nutrient metabolism in response to dietary histidine levels in juvenile largemouth bass.

Histidine played an important role in the regulation of growth, protein metabolism and lipid metabolism. Largemouth bass could regulate the protein metabolism response to different dietary histidine levels by the AAR and TOR signaling pathways. Furthermore, increasing dietary histidine level could reduce lipid accumulation by regulating PPAR*α* and PPAR*γ* signaling pathways (Figure 10), which suggested that increasing histidine supplementation might effectively alleviate fatty liver problems of largemouth bass. Based on the feed conversion rate and specific growth rate, the appropriate dietary histidine level was 1.26% of diet (2.68% of dietary protein), as determined by quadratic regression analysis.

## Figures and Tables

**Table 1:** Formulation and proximate composition of the experimental diets (%, dry matter).

Ingredient	Diet 1	Diet 2	Diet 3	Diet 4	Diet 5	Diet 6
Fish meal^1^	30	30	30	30	30	30
Rapeseed meal^1^	8	8	8	8	8	8
Soybean meal^1^	10	10	10	10	10	10
Wheat meal^1^	16	16	16	16	16	16
Fish oil	5	5	5	5	5	5
Sleeve-fish ointment	2	2	2	2	2	2
Amino acid mixes^2^	13.21	13.21	13.21	13.21	13.21	13.21
Choline chloride	0.1	0.1	0.1	0.1	0.1	0.1
Vitamin premix^3^	1	1	1	1	1	1
Mineral premix^3^	1	1	1	1	1	1
Monocalcium phosphate	2.5	2.5	2.5	2.5	2.5	2.5
Microcrystalline cellulose	3.18	3.18	3.18	3.18	3.18	3.18
Rice bran	7	7	7	7	7	7
Ethoxylquinine	0.01	0.01	0.01	0.01	0.01	0.01
Glycine	1	0.8	0.6	0.4	0.2	0
L-histidine	0	0.2	0.4	0.6	0.8	1

Compositional analysis (dry matter)						
Crude protein (%)	46.93	47.01	47.11	46.83	46.91	47.03
Crude lipid (%)	11.02	10.98	10.94	11.01	10.97	10.99
Energy (MJ/kg)	18.87	18.85	18.83	18.85	18.91	18.88
Histidine levels (%)	0.71	0.89	1.08	1.26	1.48	1.67

^1^Fish meal, rapeseed meal, soybean meal, wheat meal obtained from Wuxi Tongwei feedstuffs Co., Ltd, Wuxi, China, crude protein 65.6%, 39.2%, 39.2%, and 13.1%, respectively; crude lipid 9.5%, 6.1%, 4.3%, and 4.0%, respectively. ^2^Amino acid mixes: the amino acid content was balanced according to the amino acid composition of 47% protein level of the whole fish. The total of amino acid mixes was 13.21% including Arginine, 0.87%; Isoleucine, 0.74%; Leucine, 1.21%; Lysine, 1.40%; Methionine, 0.37%; Phenylalanine, 0.61%; Threonine, 0.74%; Valine, 0.74%; Tryptophan, 0.14%; Aspartic acid, 1.00%; Serine, 0.71%; Glycine, 1.16%; Alanine, 0.52%; Gultamic acid, 2.02%; Proline, 0.98%. All amino acids obtained from Feeer Co., LTD (Shanghai, China). ^3^Mineral premix and vitamins premix were obtained from Wuxi Hanove animal health products Co., Ltd, Wuxi, China.

**Table 2:** Amino acid composition of feed (%, dry matter).

Varieties	Diet 1	Diet 2	Diet 3	Diet 4	Diet 5	Diet 6	47% whole fish body protein composition
*Essential amino acid*							
Histidine	0.71	0.89	1.08	1.26	1.48	1.67	1.17
Arginine	2.83	2.81	2.79	2.84	2.78	2.82	2.81
Isoleucine	2.04	2.05	2.09	2.01	2.03	2.09	2.04
Leucine	3.52	3.48	3.47	3.48	3.53	3.51	3.50
Lysine	3.47	3.56	3.52	3.57	3.51	3.53	3.50
Methionine	1.12	1.13	1.11	1.14	1.09	1.11	1.11
Phenylalanine	1.97	1.94	1.93	1.95	1.96	1.94	1.95
Threonine	2.03	2.02	2.04	2.03	2.03	2.01	2.00
Valine	2.25	2.27	2.28	2.26	2.29	2.22	2.25
Tryptophan	0.49	0.51	0.49	0.48	0.50	0.51	0.50
*Nonessential amino acid*							
Aspartic acid	4.46	4.48	4.43	4.48	4.46	4.44	4.42
Serine	2.08	2.07	2.08	2.07	2.06	2.06	2.02
Glycine	4.86	4.64	4.48	4.24	4.05	3.87	3.90
Alanine	3.25	3.23	3.24	3.24	3.25	3.23	3.30
Cystine	0.25	0.27	0.23	0.25	0.26	0.25	0.27
Gultamic acid	8.48	8.42	8.47	8.43	8.41	8.42	8.50
Proline	2.61	2.62	2.61	2.62	2.63	2.61	2.64
Tyrosine	1.39	1.36	1.37	1.39	1.37	1.39	1.33

**Table 3:** The chemical analysis used in the experiment.

Items	Methods	Assay kits/testing equipment
*Composition of diets/ingredients*		
Moisture	Drying method	Electric blast drying oven (Shanghai Yiheng scientific instrument co., ltd., Shanghai, China)
Protein	Kjeldahl	Auto kieldahl apparatus: Hanon K1100 (Jinan Hanon instruments co., ltd., Jinan, China)
Lipid	Soxhlet	Auto lipid analyzer: Hanon SOX606 (Jinan Hanon instruments co., ltd., Jinan, China)
Ash	Combustion	Muffle: XL-2A (Hangzhou Zhuochi instrument co., ltd., Hangzhou, China)
Gross energy	Combustion	Oxygen bomb calorimeter: IKA C6000 (IKA WORKS GUANGZHOU, GUANGZHOU, China)
Amino acids (except tryptophan)	Acid hydrolysis	Amino acid analyzer: SYKAM S-433D (SYKAM GmbH, Munich, Germany)
Tryptophan	Alkali hydrolysis	
Liver lipid	Soxhlet	Auto lipid analyzer: Hanon SOX606 (Jinan Hanon instruments co., ltd., Jinan, China)
*Plasma parameters*		
TG^1^	International Federation of Clinical Chemistry recommended	Assay kits purchased from Mindray medical international ltd. (Shenzhen, China); Mindray BS-400 automatic biochemical analyzer (Mindray medical international ltd., Shenzhen, China).
TC^1^		

^1^TG: total triglyceride; TC: total cholesterol.

**Table 4:** Real-time PCR primer sequences used in the present study.

Target genes	Forward primer (5′-3′)	Reverse primer (5′-3′)	Sources
*TOR signaling pathway^1^*			
TOR	CCATCCTCAACCTACTTCC	CTCTCCTTCTCCTTCTTCAG	Self-designed based on transcripts
AKT	AGCGCACCTTCCATGTAGAC	GGCTATTTGCCACTTGCTGG	[30]
S6	GTAATGCAAAGGACACGGCG	GTTCCCCACCGCTCAGATAC	XP_010747297.3
4EBP1	AGCAGGAACTTTCGGTCATA	GTCAATGGGCAGTCAGAAGA	[17]
*GH-IGF-1signaling pathway^2^*			
GH	CCCCCAAACTGTCAGAACT	ACATTTCGCTACCGTCAGG	[31]
IGF-1	CTTCAAGAGTGCGATGTGC	GCCATAGCCTGTTGGTTTACTG	[31]
*AAR signaling pathway^3^*			
GCN2	GAATATCGTCCGCTACTACA	CGTCGTCGTCATCATCAT	XM_038728156.1
eIF2*α*	TAAGTCCAGCCCATCCAAAA	CACCCGAGGAGGCCATCAAG	[17]
CHOP	TGGTGGTGTTGATGGTGGTAA	AGACGTGGGGTGAGGGTGTTC	[17]
ATF4	TGGAGGTTGTTATGAAGTGT	GGAATGATGGTGGCTGTT	Self-designed based on transcripts
REDD1	TGACCTGTGTCCCTCTAATGA	ATGTGCTCCAGAAGTTTCTCA	[17]
*PPARs signaling pathway^4^*			
PPAR*α*	AGGCTTCATCACCAGAGA	TCCGCAGCAGATAATAGTAG	[30]
CPT1	TTACCGTATGGCTATGACTG	GGCTCCGATAACACCTCT	[30]
L-FABP	CTGGAGACTATTACTGGAGAG	ACACAATGCCACCAAGAG	Self-designed based on transcripts
PGC1*α*	ATGACTACAGCAGCAGAAG	TAGCCAGAGGACCAGATG	Self-designed based on transcripts
PPAR*γ*	GAGTTCTCAGTCAAGTTCAAC	AATGTAGCACCGTCTCCT	[30]
FAS	AGTTGAAGGCTGCTGATG	GCTGTGGATGATGTTGGT	[30]
SCD	CGATGCTGCTTCTTCACT	GACACGGTTCTGCCATTA	[30]
ACC	TTACATCGCAGCCAACAG	CTCTCCACCTTCCTCTACA	[30]
SREBP1	TCACCTTCCTCTTCCTCTC	TCAGTAGCCACACCAGTAT	[30]
ELOVL2	GGACACAACAATACAAGATGG	GAACAGGTAGCACAGCAAT	Self-designed based on transcripts
*Housekeeping gene^5^*			
GAPDH	ACTGTCACTCCTCCATCTT	CACGGTTGCTGTATCCAA	[30]

^1^TOR signaling pathway; TOR: target of rapamycin; AKT: protein kinase B; 4EBP1: eukaryotic initiation factor 4E binding protein 1; S6: ribosomal protein S6; ^2^GH-IGF-1signaling pathway; GH: growth hormone; IGF-1: insulin-like growth factor-1; ^3^AAR signaling pathway: GCN2: general control nonderepressible 2 kinase; eIF2*α*: eukaryotic initiation factor 2*α*; CHOP: C/EBP-homologous protein; ATF4: transcription factor 4; REDD1: DNA damage responses 1; ^4^PPARs signaling pathway: PPAR*α*: peroxisome proliferator-activated receptor *α*; CPT1: carnitine palmitoyl transferase-1; L-FABP: liver fatty acid binding protein; PGC1*α*: peroxisome proliferator activator receptor *γ* coactivator-1*α*; PPAR*γ*: peroxisome proliferator-activated receptor *γ*; FAS: fatty acid synthase; SCD: stearoyl-CoA desaturase; ACC: acetyl-CoA carboxylase; SREBP1: sterol-regulatory element binding protein-1; ELOVL2, elongation of very long-chain fatty acids 2; ^5^Housekeeping gene: GAPDH, glyceraldehyde-3-phosphate dehydrogenase.

**Table 5:** Growth performance of juvenile largemouth bass fed with the experimental diets.

Indexes	0.71%	0.89%	1.08%	1.26%	1.48%	1.67%
Initial weight (g)	12.31 ± 0.01	12.33 ± 0.02	12.33 ± 0.02	12.34 ± 0.01	12.32 ± 0.01	12.32 ± 0.01
Final weight (g)^1^	48.90 ± 2.19^a^	51.63 ± 0.37^ab^	55.48 ± 1.40^b^	55.46 ± 0.74^b^	54.31 ± 1.13^b^	51.77 ± 1.36^ab^
Weight gain rate (%)^2^	297.20 ± 17.40^a^	318.59 ± 3.52^ab^	349.88 ± 11.96^b^	349.28 ± 6.37^b^	340.70 ± 8.84^b^	320.14 ± 10.83^ab^
Feed conversion rate^3^	1.25 ± 0.05^c^	1.22 ± 0.02^c^	1.07 ± 0.02^a^	1.07 ± 0.02^a^	1.12 ± 0.02^ab^	1.18 ± 0.03^bc^
Specific growth rate (%/day)^4^	2.46 ± 0.08^a^	2.56 ± 0.02^ab^	2.68 ± 0.05^b^	2.68 ± 0.03^b^	2.65 ± 0.04^b^	2.56 ± 0.05^ab^
Protein efficiency rate^5^	1.64 ± 0.07^a^	1.68 ± 0.03^ab^	1.91 ± 0.04^c^	1.92 ± 0.03^c^	1.82 ± 0.03^bc^	1.73 ± 0.04^ab^
Feed intake rate (%)	2.65 ± 0.03^c^	2.64 ± 0.02^bc^	2.41 ± 0.04^a^	2.41 ± 0.03^a^	2.50 ± 0.02^ab^	2.58 ± 0.04^bc^
Survival rate (%)	98.89 ± 1.11	97.78 ± 2.22	98.89 ± 1.11	98.89 ± 1.11	97.78 ± 2.22	98.89 ± 1.11
Condition factor (%)	2.20 ± 0.09	2.14 ± 0.06	2.31 ± 0.13	2.23 ± 0.08	2.14 ± 0.09	2.14 ± 0.06
Viscerosomatic index (%)	7.18 ± 0.40	7.73 ± 0.83	7.00 ± 0.21	6.93 ± 0.30	7.27 ± 0.43	7.23 ± 0.06
Hepatosomatic index (%)	1.79 ± 0.14	1.61 ± 0.20	1.61 ± 0.10	1.63 ± 0.12	1.47 ± 0.22	1.39 ± 0.25
*Regressions*						
*Y*1 = −228893*x*2 + 5773.6*x* + 19.14						*R*2 = 0.7133
*Y*2 = −2*E*^+06^*x*^2^ + 46051*x* + 59.638						*R*2 = 0.7017
*Y*3 = 6366.2*x*2 − 160.23*x* + 2.0857						*R*2 = 0.7210
*Y*4 = −7645.2*x*2 + 193.18*x* + 1.4669						*R*2 = 0.7167
*Y*5 = −9927.4*x*2 + 248.81*x* + 0.3359						*R*2 = 0.7044

All data are mean value of three replicates°±°SEM. Means in the same row with different superscripts are significantly different (*P*°<°0.05). Weight gain rate (%)° = °100°×°(final body weight (g) − initial body weight (g))/initial body weight (g). Feed conversion ratio = dry feed fed (g)/wet weight gain (g). Specific growth rate (%/d) = 100 × [(Ln (final body weight (g)) − Ln (initial body weight (g)))/days]. Condition factor (%) = 100 × fish weight (g)/[body length (cm)]^3^. Hepatosomatic index (%) = 100 × (liver weight (g)/body weight (g)). Viscerosomatic index (%) = 100 × (visceral weight (g)/body weight (g)). Protein efficiency ratio (PER) = wet weight gain/protein intake. Survival rate (%) = 100 × (survival fish number/total fish number). Feed intake rate (%) = 100∗dry feed fed (g)/((total initial weight (g) + total final weight (g))/2/days).

## Data Availability

The authors confirm that the data supporting the findings of this study are available within the manuscript, tables and figures. Data are available from the corresponding author upon reasonable request.
